# Supplementary material for: An inherent T cell deficit in healthy males to C. neoformans infection may begin to explain the sex susceptibility in incidence of cryptococcosis
Source: Biol Sex Differ. 2019 Sep 2;10:44. doi: 10.1186/s13293-019-0258-2 (PMC6720413; doi:10.1186/s13293-019-0258-2)
Supplement: Supplementary file 1 — Table S1. Flow cytometry cell counts for first dataset (DOCX 20 kb) [file 13293_2019_258_MOESM1_ESM.docx]

Supplemental Table 1. Flow cytometry cell counts for first dataset.

Mean Absolute Cell Counts of Uninfected PBMCs (first dataset)

|  | CD3^+^ | CD4^+^ | CD8^+^ | CD19^+^ | Dead cells |
| --- | --- | --- | --- | --- | --- |
| Men | 20,672 | 12,121 | 5,504 | 5,520 | 4,147 |
| Women | 13,330 | 8,929 | 4,385 | 3,108 | 2,102 |

Mean Absolute Cell Counts of Infected cells (first dataset)

|  | CD3^+^ | CD4^+^ | CD8^+^ | CD19^+^ | Dead cells |
| --- | --- | --- | --- | --- | --- |
| Men | 6,964 | 3,319 | 1,855 | 1,800 | 1,897 |
| Women | 7,729 | 4,670 | 2,598 | 1,724 | 1,667 |

Fold change for Absolute Cell Counts (Infected/Uninfected, first dataset)

|  | CD3^+^ | CD4^+^ | CD8^+^ | CD19^+^ | Dead cells |
| --- | --- | --- | --- | --- | --- |
| Men | -2.97*** | -3.65*** | -2.97** | -3.07** | -2.19 |
| Women | -1.72*** | -1.91*** | -1.69 | -1.80 | -1.26 |

p < 0.05*, p < 0.01**, p < 0.001***

Supplemental Table 2. Flow cytometry cell counts for second dataset.

Mean Absolute Cell Counts of Uninfected PBMCs (second dataset)

|  | CD3^+^ | CD56^+^ | Dead cells |
| --- | --- | --- | --- |
| Men | 6,106 | 231 | 235 |
| Women | 12,576 | 843 | 582 |

Mean Absolute Cell Counts of Infected cells (second dataset)

|  | CD3^+^ | CD56^+^ | Dead cells |
| --- | --- | --- | --- |
| Men | 9,687 | 872 | 281 |
| Women | 11,248 | 964 | 469 |

Fold change for Absolute Cell Counts (Infected/Uninfected, first dataset)

|  | CD3^+^ | CD56^+^ | Dead cells |
| --- | --- | --- | --- |
| Men | +1.59* | +3.77 | +1.20 |
| Women | -1.12*** | +1.14 | -1.24 |

p < 0.05*, p < 0.01**, p < 0.001***
