# Supplementary material for: An inherent T cell deficit in healthy males to C. neoformans infection may begin to explain the sex susceptibility in incidence of cryptococcosis
Source: Biol Sex Differ. 2019 Sep 2;10:44. doi: 10.1186/s13293-019-0258-2 (PMC6720413; doi:10.1186/s13293-019-0258-2)

**An inherent T cell deficit in healthy males to *C. neoformans* infection may begin to explain the sex susceptibility in**

**incidence of cryptococcosis**

Tiffany E. Guess^1^, Joseph Rosen^1^, Natalia Castro-Lopez^2, 3^, Floyd L. Wormley

Jr.^2, 3^, Erin E. McClelland^1^

^1^Department of Biology, Middle Tennessee State University, Murfreesboro, TN, USA

^2^Department of Biology and the ^3^South Texas Center for Emerging Infectious Diseases, ^3^The University of Texas at San Antonio, San Antonio, TX, USA

Tiffany Guess: [tiffany.guess@vumc.org](mailto:tiffanyeguess@gmail.com)

Joseph Rosen: [jar8n@mtmail.mtsu.edu](mailto:jar8n@mtmail.mtsu.edu)

Natalia Castro-Lopez: [natalia.castro-lopez@my.utsa.edu](mailto:natalia.castro-lopez@my.utsa.edu)

Floyd L. Wormley Jr.: [Floyd.Wormley@utsa.edu](mailto:Floyd.Wormley@utsa.edu)

*Erin E. McClelland: [erinmcclelland91@gmail.com](mailto:erinmcclelland91@gmail.com) (Corresponding author)

**Abstract**

Background: *Cryptococcus neoformans,* the causative agent of cryptococcosis, causes ~181,000 deaths annually, with males having a higher incidence of disease than females (7M:3F). The reason for this sex bias remains unclear. We hypothesized that this disparity was due to biological differences between the male and female immune response. Methods: Peripheral blood mononuclear cells (PBMCs) from healthy donors were isolated and infected with *C. neoformans* +/-exogenous testosterone or 17-β-estradiol. *C. neoformans*, B, T, and NK cell proliferation was quantified by flow cytometry. Cytokine analysis was conducted via protein array or ELISA. Serological testing was conducted to determine previous exposure to *C. neoformans.* Results: *C. neoformans* proliferated more in male PBMCs. T cell percentages in both sexes were lower in infected versus uninfected cells. Male PBMCs had lower CD3^+^, CD4^+^, and CD8^+^ T cells percentages during infection compared to females. Cytokine profiles showed differences in uninfected male and female PBMCs, which subsided during infection. Only one donor was sero-negative for prior *C. neoformans* exposure. There was an effect of estrogen in one dataset. Conclusions: These results suggest that males show an inherent deficit in T cell response during infection, which may contribute to the increased incidence of disease in males.

**Keywords:** *Cryptococcus neoformans*, cryptococcosis, immune response, sex bias in infection, T cells, B cells, natural killer cells, estrogen, testosterone

Background

*Cryptococcus neoformans* is responsible for an estimated 220,000 cases of cryptococcosis, resulting in more than 181,000 deaths each year worldwide (1, 2). An opportunistic fungal pathogen, *C. neoformans* typically presents as pneumonia or meningitis, the latter of which is considered an AIDS-defining illness (3). Interestingly, prevalence of this disease is skewed between males and females. Numerous studies show differences in *C. neoformans* infection rates, with males having a higher incidence of disease and greater symptom severity in both HIV positive patients (8M:1F) and HIV negative patients (2-3M:1F) (4-7). Given that females are more prevalently infected by HIV (8), which significantly increases the susceptibility to *C. neoformans*, this suggests that there is a strong imbalance of *C. neoformans* disease in males. Sexual dimorphism in invasive fungal infections is not uncommon. In fact, many fungal infections occur more frequently in males. For example, males are 11 to 30 times more likely to suffer from paracoccidioidmycosis, a chronic infectious disease caused by *Paracoccidioides brasilienis*, despite similar rates of exposure to the fungus (9). However, the opposite is true of *Candida albicans* infections, which occur more frequently in women with an estimated 2F:1M split (10, 11). The differences in infection from these pathogens have been linked to sex hormones, specifically 17-β-estradiol (9, 12, 13).

Male sex is considered an independent risk factor for developing cryptococcosis (5, 14). In light of this, sexual dimorphism in *C. neoformans* infections has been the focus of a few studies; including one that examined both host and pathogen features of HIV infected patients from Botswana. Results showed that despite having increased numbers of CD4^+^ T cells, males from this patient cohort also had a higher likelihood of mortality from *C. neoformans* (6)*.* When incubated with testosterone, clinical strains showed an increased release of glucuronoxylomannan (GXM), the primary component of the *C. neoformans* capsule, suggesting that exposure to a male hormonal environment may increase the virulence of a *C. neoformans* infection (6). Clinicians in a French medical study reported more severe cryptococcosis in men including higher antigen titers and greater disseminated disease (14). Tamoxifen, an estrogen receptor antagonist, binds directly to the *C. neoformans* protein, calmodulin, blocking calcineurin activation, which results in anti-cryptococcal properties (15, 16). *In vivo* experiments using outbred mice reported higher levels of the Th1 cytokines interferon-gamma (IFN-γ) and tumor necrosis factor-alpha (TNF-α), in females compared to their male counterparts (5). Also, despite their widespread exposure to *C. neoformans* and being an immune-compromised population, cryptococcosis in children under age 16 is rare and under the age of 12 (pre-pubescent) is very uncommon (4, 17). This body of research, albeit small, suggests a relationship between *C. neoformans* pathogenesis and the hormonal environment of its host. This potential interplay necessitates further research in the context of *C. neoformans* infections and host sex.

Another variable in the pathogenesis of a *C. neoformans* infection is the host immune response, which can vary widely between individuals. Cell-mediated immunity by CD4^+^ Th1-type cells characterized by the production of IL-2, IL-6, IL-12, IFN-γ, and TNF-α is associated with the induction of protective immune responses (3, 18-21). In contrast, CD4^+^ Th2-type cell-mediated responses characterized by the secretion of IL-4, IL-5, IL-10, and IL-13 is associated with exacerbation of disease. CD4^+^ Th1-type responses induce classical macrophage activation and efficient phagocytosis and killing of *Cryptococcus* yeast (22, 23) and strong antibody-mediated immunity. The B cell response has been linked to both resistance of cryptococcosis and control of pulmonary inflammation in mice infected with *C. neoformans* (24, 25). Historically, CD4^+^ T cells have been shown to mediate fungal clearance and offer protection to the host, but recent studies describe a more complex picture implicating these T cells in advanced disease severity and higher mortality rates in both mice and HIV^+^/cryptococcosis^+^ patients (26). CD8^+^ T cells mediate direct killing of *C. neoformans* and activate macrophage responses to limit pathogen growth and survival inside macrophages (19, 22, 27). Due to the combined nature of *C. neoformans* as a potent immunomodulator and its ability to cause illness in the immune-compromised population, robust immune responses are not commonly seen (28). Given this, further investigation is needed to understand the healthy immune response to a *C. neoformans* infection, without other comorbidities affecting the outcome.

In an effort to better understand the early typical response of men and women to a *C. neoformans* infection, the present study utilized peripheral blood mononuclear cells (PBMCs) from healthy donors to correlate the immune response to a *C. neoformans* infection with host sex, providing insight into how it may affect outcome. The aim of this study was to begin to unravel the complexity of sex-specific, host-pathogen interactions by examining the effect of host sex and sex hormones on host immunity to *C. neoformans.* We hypothesized that males have an increased incidence of disease due, at least in part, to differences in the immune response during a *C. neoformans* infection.

Methods

*Volunteers*

This study was carried out in the Biology Department of Middle Tennessee State University in Murfreesboro, TN. For the first dataset, 43 healthy volunteers (22 male:21 female) aged ≥ 18 years were prospectively enrolled. Of those 43, 40 (21 male:19 female) samples were utilized. For the second set of experiments, forty volunteers (19 male:21 female) ≥ 18 years were prospectively enrolled. Of those 40, 28 (14 male:14 female) samples were utilized. Subjects were excluded if they yielded < 15 mL of blood due to the number of cells needed for these experiments.

*Peripheral Blood Mononuclear Cell (PBMC) Isolation*

Peripheral blood samples were obtained from volunteers by a licensed phlebotomist via venipuncture. Approximately 25 mL of blood per person was collected in EDTA collection tubes (BD Biosciences, Franklin Lakes, NJ) and labeled with a unique identifier. Heparanized blood was overlayed onto room temperature Ficoll (Sigma Aldrich, St. Louis, MO) in a 1:1 ratio in a sterile 50 mL conical tube. Samples were centrifuged at 400 x *g* for 30 minutes at ambient lab temperature (~ 21°C) with the brake off to ensure that deceleration did not disrupt the density gradient. Two mL of sera from each subject’s sample was stored at -80^o^C for subsequent antibody-reactivity testing. The buffy coat, containing the mononuclear cell layer, was extracted using a transfer pipette and washed three times with lipopolysaccharide (LPS)-free phosphate buffered saline (PBS). Cells were resuspended in RPMI 1640 medium (Sigma, St Louis, MO) plus 10% human serum (50:50 male:female, Innovative Research, Novi, MI) and counted on a hemocytometer using a 1:1 Trypan blue (Sigma Aldrich, St. Louis, MO) exclusion (0.4% solution). Aliquots of 2 x 10^8^ viable cells/2 mL were cryopreserved using a method described previously (29) and infected at a later date.

*MOI Determination*

To establish the appropriate multiplicity of infection (MOI), preliminary experiments were conducted using PBMCs infected with *C. neoformans* using a variety of MOIs: 1:1, 1:10, 1:50, 1:100, and 1:1,000. The cells were incubated in a 37^o^C CO_2_ incubator and assessed via flow cytometry after seven days (30). The 1:100 MOI was determined to be the most beneficial ratio as it stimulated proliferation but did not cause significant death of the PBMCs (< 15%) (data not shown).

*PBMC Infection*

Cryopreserved PBMCs were removed from liquid nitrogen and thawed in a 37^o^C water bath. After thawing, cells were added to 10 mL warm RPMI 1640 medium (Sigma Aldrich, St. Louis, MO) plus 10% human serum (50:50 male:female, Innovative Research, Novi, MI) and centrifuged at 400 × *g* to pellet PBMCs. Cells were resuspended in 500 μL warm RPMI and counted on a hemocytometer using a 1:1 trypan blue exclusion. One hundred μL of 5 x 10^6^ viable cells/mL PBMCs were seeded in 96-well round-bottomed plates and infected with the wildtype *C. neoformans* strain, H99S (Dr. John Perfect, Duke University, (31)) at a concentration of 5 x 10^4^ cells/mL in 100 μL RPMI (1:100 MOI) and allowed to incubate for seven days in a 37^o^C CO_2_ incubator.

In an effort to determine whether the addition of exogenously added sex hormones would affect the immune response, physiological levels of testosterone (10 ng/mL, Sigma Aldrich, St. Louis, MO) or 17 β-estradiol (400 pg/mL, Sigma Aldrich, St. Louis, MO) were added to subsets of infected and uninfected PBMCs on day one of incubation. Separate PBMC and *C. neoformans* negative controls were incubated with RPMI only. All samples were set up in triplicate. One hundred μL warm RPMI was added to each well on day four of incubation to refresh nutrients. After seven days, PBMC culture supernatants were harvested, frozen, and stored at -80 ^o^C for subsequent cytokine testing. PBMCs were resuspended in LPS-free PBS and incubated with fluorescent antibodies (**Tables 1 and 2**) for 20 minutes at approximately 21^o^C (lab temperature), in the dark. After incubation, PBMCs + antibodies were centrifuged at 400 × *g* for five minutes, the supernatant discarded, and the cells resuspended in 300 µL PBS for flow cytometry acquisition (**Fig. 1**).

**Table 1.** Flow cytometry reagent list for the first dataset.

| **Antibody/Probe** | **Cell Expression** | **Fluorochrome** | **Emission Spectra (nm)** | **Vendor** |
| --- | --- | --- | --- | --- |
| Anti-human CD3 | Pan expression T cell surface marker | APC | 650/660 | BD Biosciences (Franklin Lakes, NJ) |
| Anti-human CD4 | Helper T cell surface marker | PE | 496/578 | BD Biosciences |
| Anti-human CD8 | Cytotoxic T cell surface marker | FITC | 494/520 | BD Biosciences |
| Anti-human CD19 | Pan expression B-cell surface marker | PC7 | 496/785 | Beckman Coulter (Brea, CA) |
| Live-dead discriminator | Compromised cell membrane marker | 7AAD | 546/647 | Thermo Fisher (Waltham, MA) |

Abbreviations: CD, cluster of differentiation; APC, allophycocyanin; PE, phycoerythrin; FITC, fluorescein isothiocyanate; PC7, phycoerythrin cyanine 7; 7AAD, 7-aminoactinomycin D.

**Table 2.** Flow cytometry reagent list for the second dataset.

| **Antibody/Probe** | **Cell Expression** | **Fluorochrome** | **Emission Spectra (nm)** | **Vendor** |
| --- | --- | --- | --- | --- |
| Anti-human CD3 | Pan expression T cell surface marker | PC7 | 496/785 | BD Biosciences (Franklin Lakes, NJ) |
| Anti-human CD56 | Natural killer cell surface marker | PE | 496/578 | BD Biosciences |
| *C. neoformans* antibody 18B7 | IgG to *C. neoformans* capsule | AF488 | 490/525 | A kind gift of Dr. Arturo Casadevall |
| Live-dead discriminator | Compromised cell membrane marker | PI | 493/636 | Molecular Probes |

Abbreviations: CD, cluster of differentiation; PC7, phycoerythrin cyanine 7PE; PE, phycoerythrin; AF488, AlexaFluor 488; PI, propidium iodide.

*Flow Cytometry Acquisition and Analysis*

A Guava® easyCyte 8HT was used for flow cytometry. A forward scatter threshold of 800 was used to avoid most debris in counting; 30,000 events/well were collected in all wells with the exception of *C. neoformans* only controls where 5,000 events/well were collected. For the first dataset, due to the flow cytometer being shared between labs and the settings being changed without our knowledge, a subset of samples had 100,000 events collected, which unfortunately skewed the cell counts for this dataset (Supplemental Table 1). The cell counts for the second dataset are depicted in Supplemental Table 2. Compensation was performed using single-color controls of uninfected PBMCs. Analytic gating of flow cytometry data was analyzed using InCyte version 3.1. For multi-color analysis, lymphocytes were identified in the same manner, and standard elliptic gates were applied to all samples. B (CD19^+^) and T (CD3^+^, CD4^+^, CD8^+^) cell proliferation as well as dead cells were measured in both uninfected and infected PBMCs for the first dataset. Due to the limitations of how many markers our flow cytometer could measure at once, we repeated the experiment with different donors and measured T cell (CD3^+^), natural killer (NK) cell (CD56^+^), and *C. neoformans* proliferation (mAb 18B7^+^), as well as dead cells in both uninfected and infected PBMCs. We averaged the counts of *C. neoformans* in each of the treatments, calculated the percent *C. neoformans*/treatment and subtracted that percentage from the total cell counts from the first dataset to determine total lymphocyte counts. Representative dot plots for a male and female donor are illustrated in **Figures 2** and **3**, respectively.

In Situ *Cytokine Analysis*

Cytokine profiles were analyzed using cell supernatants after seven days of incubation. Supernatants from PBMC incubations from the first dataset with or without *C. neoformans* were thawed and assayed for the presence of IL-2, IL-4, IL-6, IL-8, IL-10, IL-12, granulocyte-macrophage colony-stimulating factor (GM-CSF), TNF-α, and IFN-γ. All cytokines with the exception of IL-12 were analyzed using the Bio-Plex protein array system (Luminex-based technology; Bio-Rad Laboratories, Hercules, CA). The human IL-12p70 BD-OptEA kit (BD Biosciences) was used to measure the production of IL-12 as per the manufacturer's instructions. The detection limit of the assay was 7.8 pg/ml as stated by the manufacturer. Because there were no significant differences noted, cytokine analysis was not conducted on the cell supernatants from the second dataset.

*C. neoformans Serology Testing*

The serology status of donors was assessed using the YA Crypto Antibody Tube Agglutination System (Immy, Norman, OK), which detects *C. neoformans* antibodies in human serum (32). To further confirm serology results, all negative agglutination samples were tested against immunoblots of H99S cell lysate (31). Each lane contained a 1:100 dilution of donor sera. A positive donor was used as a positive control and Tris-buffered saline alone was used as a negative control on each membrane. A 1:2000 dilution of goat anti-human IgG-HRP (ThermoFisher) was added as the secondary antibody. The blot was visualized using chemiluminescence. Any lane that had < 2 bands was scored negative and > 2 bands was scored positive as done in Chen et al. (33) (data not shown). Sera from 68 of the 74 donors were tested. Due to equipment failure, the sera of six donors were compromised and unable to be assessed.

*Statistical Analysis*

*C. neoformans,* B, T, and NK cell, and dead cell data were analyzed using JMP, version 14 (SAS Institute). Variables were compared across groups, using multivariate analysis of variance (MANOVA). Because a MANOVA controls for an increase in type I error, this was followed by simple contrasts to determine which cell marker was responsible for the effect. Cytokine data was analyzed using a two-way analysis of variance (ANOVA) and Bonferroni Correction to correct for multiple comparisons. Categorical variables were analyzed using a χ^2^ test. Fold changes were calculated by dividing the mean percentage of infected cells for each marker by the mean percentage of uninfected cells using the corresponding marker. Statistical significance was defined as *p*-value of < 0.05.

**Results**

In situ *cytokine analysis and CD25^+^ cells: Male PBMCs are proliferating*

The first step was to determine if infected PBMCs were actually proliferating in response to infection with *C. neoformans*. Thus, we compared cytokine levels at day 7 between uninfected and infected PBMCs. In infected male PBMCs, we saw significant increases in the levels of the cytokines IL-2, IL-4, IL-6, IFN-γ, and TNF-α (*p* < 0.05 for all cytokines), suggesting that the PBMCs were proliferating (**Fig. 4A**). However, there was no difference in cytokine levels between uninfected and infected female PBMCs (data not shown). We also measured CD25 levels, an activation marker for CD4^+^ T cells (34), for a small subset of donors in the first dataset (**Fig. 4B**). While the data were not significant, likely due to the small sample size, the number of CD25^+^ cells in infected male and female PBMCs increased compared to uninfected PBMCs. Taken together, the data strongly suggest that male PBMCs were proliferating in response to infection, while the data for proliferation in infected female PBMCs were less clear.

*Immune cell response in uninfected cells: Lower percentages of CD3^+^ and CD4^+^ T cells in men*

We next wanted to establish any differences in uninfected male and female immune cells to ensure that changes seen during infection were due to *C. neoformans* and not variations caused by sex alone. Due to the limitations of how many markers our flow cytometer could measure at once, we conducted two independent experiments. The first experiment measured B (CD19^+^) and T (CD3^+^, CD4^+^, CD8^+^) cell proliferation as well as dead cells in both uninfected and infected PBMCs. The second experiment measured T cell (CD3^+^), natural killer (NK) cell (CD56^+^), and *C. neoformans* proliferation (mAb 18B7^+^), as well as dead cells in both uninfected and infected PBMCs. In the first dataset, males had a lower percentage of CD3^+^ cells, a surface marker expressed on all T cells, compared to females (53.4% vs. 60.9%, F=8.17, df=1770, *p* = 0.004). However, this difference is typical of healthy men and women according to previously published reference ranges (35-37). Males also had lower percentages of CD4^+^ helper T cells compared to females (34.8% vs. 41.6%, F=6.76, df=1770, *p* = 0.009). Amounts of CD8^+^ cytotoxic T cells, and B cells, as measured by CD19, a marker expressed on the surface of all B cells, were all approximately the same in uninfected control cultures from men and women (*p* > 0.05). Numbers of dead immune cells after seven days of culture were also similar between sexes (**Fig. 5**). The second dataset showed the same pattern in CD3^+^ expression between men and women, with men having lower percentages of CD3^+^ cells (69.9% vs. 81.6%, F=17.90, df=738, *p* < 0.0001). The numbers of NK cells and dead immune cells after seven days of culture were similar between sexes (**Fig 6**). There were no differences in immune cell numbers in either sex in subsets of cells with exogenously added hormones (data not shown).

*C. neoformans proliferation during infection: Increased proliferation in male vs. female PBMCs, except with the addition of 17-β-estradiol*

To discern any differences in *C. neoformans* proliferation in the presence of immune cells from healthy men and women, we quantified yeast numbers via flow cytometry after seven days incubation with PBMCs. While there was no difference in proliferation in male or female PBMCs with a specific hormone treatment, when *C. neoformans* counts were compared between men and women, *C. neoformans* generally proliferated better in PBMCs from men compared to women, except when exogenous 17-β-estradiol was added. Male PBMCs had significantly higher cell counts in treatments without hormone (F=6.20, df=246, *p* = 0.013) and with the addition of exogenous testosterone (F=4.53, df=246, *p* = 0.034), but there was no difference in cell counts between males and females when exogenous 17 β-estradiol was added (**Fig. 7**). These data suggest that *C. neoformans* replicates more readily in a male environment and that 17 β-estradiol may inhibit proliferation in male PBMCs.

*Effect of C. neoformans on the immune response: Decreased cell percentages after infection*

*C. neoformans* is a known immunomodulator (38, 39). Thus, we wanted to understand if any immune cells were depressed during infection. To do this, we calculated the fold change between uninfected and infected PBMCs in all markers and both sexes (**Tables 3** **and** **4**). In the first dataset, compared to uninfected cells, PBMCs infected with *C. neoformans* showed significantly decreased percentages of all three measured T cell markers in both male (CD3^+^: 53.4% vs. 16.9% F=320.6, CD4^+^: 34.8% vs. 9.8%, F=151.3, CD8^+^: 16.1% vs. 4.7%, F=31.5, *p* < 0.0001 & df=3540 for all markers) and female cell populations (CD3^+^: 60.9% vs. 28.5%, F=229, CD4^+^: 41.6% vs. 17.2%, F=130.5, and CD8^+^: 19.7% vs. 9.4%, F=23.1, *p* < 0.0001 & df= 3540 for all markers). Like T cells, B cell percentages declined in male (CD19^+^: 14.6% vs. 5.4%, F=25.6, df=3540, *p* < 0.0001) and female (CD19^+^: 14.2% vs. 6.4%, F=9.55, df=3540, *p* = 0.002) samples. Interestingly, compared to uninfected cells, the percentages of dead PBMCs decreased during infection in males (10.0% vs. 4.4%, F=7.67, df=3540, *p* = 0.005) but not females (7AAD^+^: 8.7% vs. 6.0%, F=1.57, df=3540, *p* > 0.05).

**Table 1.** Fold changes of cell proliferation in the first dataset during a *C. neoformans* infection. PBMCs from healthy men and women were incubated for 7 days with or without *C. neoformans* and cell numbers were measured via flow cytometry. Fold changes were calculated by dividing the mean percentage of infected cells for each marker by the mean percentage of uninfected cells using the corresponding marker, *p* < 0.05*, *p* < 0.01**, *p* < 0.001***

|  | CD3 | CD4 | CD8 | CD19 | Dead Cell Stain |
| --- | --- | --- | --- | --- | --- |
| Men | -3.16*** | -3.57*** | -3.43*** | -2.87*** | -2.27** |
| Women | -2.14*** | -2.42*** | -2.10*** | -2.05** | -1.45 |

**Table 2.** Fold changes of cell proliferation in the second dataset during a *C. neoformans* infection. PBMCs from healthy men and women were incubated for 7 days with or without *C. neoformans* and cell numbers were measured via flow cytometry. Fold changes were calculated by dividing the mean percentage of infected cells for each marker by the mean percentage of uninfected cells using the corresponding marker *p* < 0.05*, *p* < 0.01**, *p* < 0.001***

|  | CD3 | CD56 | Dead Cell Stain |
| --- | --- | --- | --- |
| Men | -1.19*** | -1.55 | +1.04 |
| Women | -1.26*** | +1.36 | -1.33 |

Similar to the first dataset, the second dataset showed a depression of T cells measured during infection in samples from men (CD3: 69.8% vs. 58.6%, F=17.9, df=1476, *p* < 0.0001) and samples from women (CD3: 81.6% vs. 64.8%, F=39.8, df=1476, *p* < 0.0001). Compared to uninfected PBMCs, the NK cell percentage remained the same in samples from men (CD56: 11.4% vs. 7.3%, F=2.28, df=1476, *p* > 0.05) and in samples from women (CD56: 6.3% vs. 8.6%, F=0.73, df=1476, *p* > 0.05) in the presence of *C. neoformans.* Percentages of dead cells also remained the same in men (2.6% vs. 2.7%, F=, df=1476, *p* > 0.05) and women (3.8% vs. 2.9%, F=0.12, df= 1476, *p* > 0.05). These results confirm that, overall, *C. neoformans* depresses the immune response, even in proliferating cells from healthy individuals. Similar results were seen in subsets of cells incubated with exogenous sex hormones (data not shown).

*Differences in the male and female immune cell response to C. neoformans infection: Males have lower percentages of all T cell markers compared to females*

After establishing baseline differences in immune cells, *C. neoformans* proliferation, and *C. neoformans* effects on the immune response, we were now ready to determine whether features of the adaptive immune response to *C. neoformans* differed between healthy males and females, given that males have a higher incidence of both disease and death from cryptococcosis (6, 40-44). In the first dataset, men had strikingly lower percentages of all T cell markers measured compared to women at 7 days post *C. neoformans* infection (CD3^+^: 16.9% vs. 28.5%, F=30.7; CD4^+^: 9.8% vs. 17.2%, F=12.6; and CD8^+^: 4.7% vs. 9.4%, F=5.22, *p* < 0.02 & df=3540, for all markers). The original differences between CD3^+^ and CD4^+^ observed in PBMCs from males and females grew significantly during infection (*p* = 0.0002, *p* < 0.0001, respectively). There was no significant difference between male and female B cell percentages in the presence of *C. neoformans* (5.5% vs. 6.3%, F=0.14, df=3540 *p* > 0.05) (**Fig. 8**). Further, there were no differences in dead cell percentages (4.4% vs. 6.0%, F=0.63, df=3540, *p* > 0.05) or in the immune response in cell subsets with exogenously added testosterone or 17 β-estradiol (data not shown). Like the first dataset, the second dataset showed significantly lower percentages of T cells in infected male compared to infected female samples (CD3^+^: 58.6% vs 64.8%, F=5.83, df=738, *p* < 0.016). In this dataset as well, original differences between the percentages of CD3^+^ T cells observed in PBMCs from males and females grew significantly during infection (*p* < 0.0001). There were no differences in NK cells (CD56^+^: 7.3% vs. 8.6%, F=0.24, df=738, *p* > 0.05) (**Fig. 9)** or dead cells (2.7% vs. 2.9%, F=0.005, *p* > 0.05) between sexes (data not shown). This data reveals lower percentages of T cells in healthy male PBMCs during a *C. neoformans* infection.

*Correlation of the immune response and estrogen: Females with increased circulating estrogen had increased CD3^+^ T cells and CD56^+^ NK cells during infection*

Interestingly, there was a significant effect of estrogen between infected cell percentages for males and females (F=21.05, df=738, *p* < 0.0001) in the second dataset. To determine if this was due to circulating levels of estrogen in the female donors, we tested whether there was a difference depending on the week of the menstrual cycle the donors were in when they donated blood. Of the 14 women volunteers in the second dataset, 6 of them were in the second week of their cycle, which is known to have higher levels of circulating estrogen (45). Female donors in week 2 of their cycle had significantly higher percentages of CD3^+^ (F=7.44, df=342, *p* = 0.0067) and CD56^+^ (F=12.6, df=342, *p* = 0.0004) cells compared to donors that were in weeks 1, 3, and 4 of their cycle. When we looked for differences in cell percentages in uninfected female samples based on the week of their menstrual cycle, the pattern was different. Uninfected PBMCs from female donors in week 3 of their cycle had significantly higher percentages of CD3^+^ (F=11.8, df=342, *p* = 0.0007) and CD56^+^ (F=5.40, df=342, *p* = 0.02) cells compared to donors that were in week 0 of their cycle (data not shown). These data suggest that circulating levels of estrogen can affect immune cell proliferation.

*Cytokine profiles induced by C. neoformans infection of PBMCs: Lower Th1 cytokines in males before infection; no difference between the sexes after infection*

During a *C. neoformans* infection, the expression of Th1 rather than Th2 cytokines is instrumental in mounting an effective response against the pathogen (19). To determine whether production of these molecules were different between men and women, cytokine expression was measured in uninfected and infected cell supernatants from the first dataset at day seven. Cytokines measured include: IL-2, IL-6, IL-12, IFN-γ, TNF-α, and GM-CSF, which are indicative of a Th1 response. Expression of IL-4, IL-5, and IL-10 was also measured and are seen during a Th2 cytokine response (**Fig. 10**). In uninfected supernatants, male donors had significantly lower levels of IL-2, IL-4, IL-6, IFN-γ, and TNF-α compared to their female counterparts (*p* < 0.05 for all markers). There were no differences in IL-8, IL-12, and GM-CSF levels between uninfected cell supernatants in males and females, nor were there any differences in cytokine levels in males and females in infected cell supernatants. Comparing cytokine profiles between uninfected and infected groups (both males and females combined), we saw significant increases in the Th1 cytokines IL-2, GM-CSF, IFN-γ, and TNF-α (*p* = 0.021, 0.011, 0.004, 0.016, respectively), and IL-4 (*p* = 0.047), a Th2 cytokine, during a *C. neoformans* infection. There were no differences in IL-6, IL-12, and IL-10 levels between uninfected and infected cells (**Fig. 11**). Given that there were no differences in the measured immune response of cells treated with sex hormones, supernatants from these groups were not analyzed. Further, the cytokine expression from the second dataset was not analyzed as there were no differences in cytokine expression in infected cells from the first dataset.

*C. neoformans antigenic testing: Males and females have equal exposure*

Historically, it has been postulated that males suffered greater incidences of cryptococcosis due to more frequent environmental exposure of *C. neoformans* compared to females, particularly in rural areas (46). However, recent studies have shown relatively equal exposure rates between men and women (47, 48). To better understand our donor cohort, *C. neoformans* antigenic testing was performed on sera collected from subjects in both datasets to determine serology status of each, using an agglutination test (Immy) and confirmed by immunoblot. Only one donor had a history of working with *C. neoformans.* Of the 62 apparently healthy subjects tested (40 from the first dataset and 22 from the second), 35 were male (56.4%), and the mean age was 24.7 years. Only one sample was confirmed as sero-negative (1.61%) through immunoblotting. The sero-negative donor was a healthy female. Since previous *C. neoformans* exposure should generate an enhanced memory response, the immune response was correlated to serology status. There was a significantly higher percentage of CD3^+^/CD4^+^ T cells in the sero-negative donor compared to the sero-positive donors (58.3% vs. 26.0%, F=15.5, df=1770, *p* < 0.0001). There were no differences in CD3^+^/CD8^+^ T cell percentages, B cell percentages, or cytokine response (data not shown). As there was only one donor without previous exposure to *C. neoformans*, this data should be interpreted with caution.

**Discussion**

This is the first study to look at the adaptive immune responses of healthy male and female PBMCs when exposed to *C. neoformans* in the absence or presence of testosterone or 17 β-estradiol. The goal was to understand the differences in the host immune response between males and females to determine if those differences could contribute to the observed differences in cryptococccosis between the sexes. Our cohort of subjects is unique, in that, they are healthy and not typical candidates for cryptococcal disease. Given the nature of cryptococcosis, it is accepted that patients suffering from the disease have multiple comorbidities, or at the very least, a compromised immune system, making it difficult for researchers to discern how *C. neoformans* acts on the host devoid of other variables. Keeping this in mind, these experiments were designed with the aim of teasing apart differences that may be masked or accentuated when studying typical *C. neoformans* patients.

The *in situ* cytokine levels and amounts of the activation marker CD25 between uninfected and infected male and female PBMCs were measured to determine if the PBMCs were proliferating in response to infection with *C. neoformans*. The cytokine data clearly suggest that male PBMCs, but not female PBMCs, were proliferating in response to infection. Additionally, levels of CD25^+^ cells increased in infected male and female PBMCs, but the increases were not significant, likely due to the small sample size. It is interesting that the cytokine data suggest that male PBMCs, but not female PBMCs, were proliferating, especially since we saw significantly lower percentages of all tested T cells in male PBMCs compared to female PBMCs after infection. We hypothesize that since females naturally have higher levels of CD3+ and CD4+ T cells (35-37), perhaps there were enough circulating T cells in the PBMCs to control the infection.

Notably, *C. neoformans* proliferated better in PBMCs isolated from males compared to females, both without additional hormone and in the presence of testosterone. Interestingly, there was no difference in *C. neoformans* cell counts between males and females in the presence of added 17 β-estradiol, suggesting that 17 β-estradiol may either inhibit proliferation or boost the immune response, as observed by Mohr et al (49). Given our previous data showing increased proliferation of *C. neoformans* in male macrophages *ex vivo* (6), we hypothesize that male immune cells cannot efficiently contain fungal proliferation, hence the increased proliferation in ethanol and testosterone. However, with the addition of 17 β-estradiol and its known stimulation of the immune system (50-52), we hypothesize that 17 β-estradiol is having a larger impact on the efficiency of male immune cells, since they are not normally exposed to high levels of 17 β-estradiol, and thus *C. neoformans* proliferation in males is decreased in the presence of 17 β-estradiol. Since 70% of patients with cryptococcal disease are male (5, 14, 53), this data suggests that the male immune response is less efficient than the immune response of females at controlling a *C. neoformans* infection.

This idea was reinforced by the T cell data in males. Reference ranges for healthy adults in the U.S. show that CD3^+^ and CD4^+^ counts are typically higher in females (36, 37). Uninfected PBMCs served as the negative control in this study and reflected those differences in the CD3^+^ and CD4^+^ population. During infection, T cell percentages in both males and females dropped significantly compared to uninfected controls. This is unsurprising given *C. neoformans* known immunomodulatory effects (28, 54-56). However, even though male PBMCs were proliferating in response to infection, all measured T cell markers were significantly lower in males than females in infected groups, suggesting a weakened T cell response in males in the presence of *C. neoformans*. This finding could also be due to differences in the phosphorylation state of SAMHD1 during *C. neoformans* infection, as the authors found that sex influenced the phosphorylation state of SAMHD1 during HIV-1 infection, which translated into decreased macrophage susceptibility in females (57).

A robust cytotoxic T cell (CD8^+^) response is needed to successfully mediate the immune response to a cryptococcal infection and has been shown to directly inhibit the growth of *C. neoformans* (58, 59). The helper T cell (CD4^+^) response is more complex. Despite their ability to decrease fungal burden in the host, CD4^+^ cells have recently been linked to advanced clinical symptoms and mortality (26, 60). One such study examined HIV^+^/cryptococcal meningitis^+^ patients and found that despite having higher CD4^+^ T cells, males had higher risk of death (6). In HIV^+^ patients, T cell depletion is the primary predisposing cause for development of cryptococcal meningitis (61). Given this, our results suggest an inherent T cell deficit in the male immune response when confronted with *C. neoformans.* This may begin to explain why males experience higher rates of disease and death from cryptococcosis.

While T cells represent one arm of the adaptive immune response, B cells represent the other. During infection, B cell (CD19^+^) percentages fell significantly in cell populations from both men and women, but there was no difference between the two groups in the presence of *C. neoformans*, suggesting that the significant drop in T cells observed in men may be more important than B cells in an immune response to *C. neoformans*. The role B cells play in a *C. neoformans* infection have not been as well elucidated as those of T cells, but multiple studies report the necessity of B cells, often serving as a first line of defense during *C. neoformans* infections and a correlation between increased levels of B cells (IgM^+^) and a decreased likelihood of immunocompromised patients developing cryptococcosis (25, 62, 63). It should be noted, however, that most of these studies have taken place with HIV^+^ subjects that already have T cell deficiencies and therefore may rely more heavily on an effective B cell response.

To further understand the immune response to *C. neoformans* in healthy cell populations, cytokine analysis was conducted using supernatants from the first dataset. Interestingly, uninfected cell supernatants from males showed lower levels of both the Th1 cytokines IL-2, IFN-γ, TNF-α and the Th2 cytokines IL-4, and IL-6 compared to uninfected female supernatants. During infection, however, the differences in cytokine expression between males and females disappeared, which corresponds to another recent study that quantified the healthy host response to *C. neoformans* (64). There were increases in IL-2, IL-4, GM-CSF, IFN-γ, and TNF-α in infected supernatants as a whole (males and females combined) compared to uninfected samples, which is indicative of a healthy response consisting of mostly Th1 cytokines (18, 65, 66). Given that IL-2 is synthesized by activated T cells (67), it is perplexing that during infection, IL-2 levels were the same between the sexes even though male PBMCs were clearly proliferating. This could be due to the lack of proliferation of female PBMCs. It is also possible that male cell populations included higher numbers of NK cells, which are activated in the presence of IL-2 and have limited anti-cryptococcal properties (59, 68). Because of this possibility, we measured amounts of NK cells during infection in the second dataset. While uninfected male PBMCs had higher percentages of NK cells compared to females, there was no difference during infection, perhaps due to the similar levels of IL-2 between the sexes. The lower levels of cytokine expression in uninfected male supernatants, but the ability to overcome those deficits during infection, paints a complex picture and does not point to a definitive cytokine profile between the sexes. This is a healthy cohort, however, and may be more suggestive of an ideal immune response to *C. neoformans* than anything else.

Prior to the HIV epidemic, it was postulated that males had a higher incidence of cryptococcosis due to higher exposure rates, particularly in rural areas (46, 69). More recent studies show that not to be the case, with similar exposure rates for males and females, suggesting biological reasons for the sex bias in *C. neoformans* infections (47, 70). To better understand the donor population in this study, antigenic testing was conducted to determine serological status. Of the 62 subjects tested, 61 (98.4%) demonstrated immune reactivity to cryptococcal antigens indicating previous exposure, 28 males and 33 females. This study was performed in Murfreesboro, TN, a city with approximately 130,000 residents (71).

Many factors are thought to influence the differences between male and female immune responses. Environmental stimuli, microRNAs, and X-linked immunoregulatory genes are all evidenced to contribute to what is commonly considered the female immune advantage (72-74). The majority of research in sexual dimorphism between males and females during an immune challenge, however, is focused on sex hormones (50-52, 72, 75, 76). Testosterone has anti-inflammatory properties, whereas estrogen exhibits pro-inflammatory properties (50-52). Therefore, when designing this study, we hypothesized that overlaying exogenous levels of sex hormones onto PBMCs would exaggerate the immune response. For the purposes of this study, we chose normal/high-range hormone concentrations to overlay onto PBMCs given that the range of sex hormone levels considered normal is vast. For healthy males, age 20-29, testosterone levels vary between 0.8 -11 ng/mL, and in females, 17 β-estradiol falls between 20-430 pg/mL with the levels fluctuating widely throughout the menstrual cycle (35, 77). Thus, we expected that added levels of 17 β-estradiol would increase B and T cell proliferation, and added levels of testosterone would show a corresponding decrease. There was, however, no change in B and T cell reactions in the presence of added sex hormones in both the infected and uninfected groups for the first dataset. There was a significant difference of cell percentages between males and females with added 17 β-estradiol in the second study, likely due to the number of female donors that had higher levels of circulating estrogen when they donated blood. This data, in combination with the increased *C. neoformans* proliferation in male PBMCs suggests two possibilities: 1) that levels of circulating sex hormones can actively affect an immune response, and 2) higher levels of 17 β-estradiol may boost the immune response. The known pro-inflammatory effects of 17 β-estradiol may explain both this data and the data of Mohr et al (49, 69).

The key strength of our study is the large number of healthy donors (68 in total). To our knowledge, this study is the largest reported to date evaluating the healthy immune response to *C. neoformans* in men and women, thus increasing the reliability of our findings. There are limitations in this study worth noting. First, these experiments were done in healthy individuals who normally do not develop cryptococcal disease. Thus, in order for the observed differences in the male and female immune response to contribute to the future understanding of cryptococcal disease, the differences would have to result in important changes in immune function that would persist in relevant ‘at risk’ populations. Future studies will examine this hypothesis. Second, since these studies were conducted on *ex vivo* cells, they may not have contained the full cellular milieu found within an intact organism, which possibly limited the effect. However, the fact that we saw differences between the T cell responses in PBMCs from males vs. females *ex vivo* indicates that enough of the cellular milieu remained to have an effect. In addition, the cells were grown in media and FBS that did not have estrogens removed (78, 79). Thus, the cells were likely exposed to higher baseline levels of hormones, which may have limited the effects of the added exogenous hormones. Third, equipment availability forced us to split our analysis into two datasets, which allowed for only a partial immune cell analysis in each set of experiments. Ideally, this work would be repeated analyzing the response of all immune cells in the presence of *C. neoformans*, simultaneously. Fourth, we consistently saw a decrease in the percentages of the immune cells measured in the presence of *C. neoformans*. This is unsurprising given the immunomodulatory effects of *C. neoformans*; however, we did not see a corresponding increase in any of the cell markers measured. This is possibly due to a proliferation in unmeasured immune cells (macrophages, neutrophils, eosinophils, basophils); PBMCs damaged due to infection such that the flow cytometer was unable to properly detect them; or most likely, a combination of both. However, when quantifying B cells, T cells, NK cells, and *C. neoformans* during infection, we were able to account for approximately 88-95% of the total cell population collected, suggesting that any remaining unmeasured immune cells are a small percentage of the population. Finally, the age of our donor population was relatively young (24.7 years). We know that age plays an important role in the immune response of both men and women; therefore, results of similar testing may be different in both pre-pubescent and post-menopausal populations. Future studies should include immune cell analysis of different age groups as well as samples from immune compromised individuals and diseased *in-vivo* models.

**Perspectives and Significance**

During a *C. neoformans* infection of healthy male and female PBMCs, all percentages of measured T cell markers were significantly lower, and *C. neoformans* proliferated in greater numbers in the presence of male immune cells compared to female immune cells. These results, in combination with the similar response of B cell and NK cell proliferation in this study and in intracellular fungal proliferation in macrophages as observed in another study (64), between the sexes points to an inherent deficiency of male T cells to effectively combat a *C. neoformans* infection. Looking at the immune response of healthy individuals reveals differences that may otherwise be overlooked. At present it is not clear what role testosterone and 17 β-estradiol play in the immune response to *C. neoformans*, but the data suggest they do play a role*.* In total, these results indicate that the difference in outcomes of cryptococcosis between men and women is biologic, can be at least partially explained by levels of circulating sex hormones and the adaptive immune response, and cannot simply be chalked up to differences in exposure rates. These experiments are an important first step in elucidating the biological sex differences seen in response to a *C. neoformans* infection and hopefully lay the groundwork for future research that uncovers the mechanisms behind these differences.

**Declarations**

*Ethics Approval and Consent to Participate*

The study design and protocol were approved by the Institutional Review Board (IRB) of Middle Tennessee State University under protocol # 15-301. All participants provided written informed consent.

*Consent for Publication*

Not applicable.

*Availability of Data and Material*

The datasets generated and/or analyzed during the current study are available from the corresponding author on reasonable request.

*Competing Interests*

The authors declare no competing interests.

*Funding*

This research was supported by the Molecular Biosciences Program at Middle Tennessee State University and 2RO1AI071752 (F.L.W. Jr.) from the National Institute of Allergy and Infectious Diseases (NIAID).

*Author’s Contributions*

Conceptualization, EM; Data curation, TG, JR, and NC-L; Formal analysis, TG, NC-L, FW, and EM; Methodology, TG, JR, EM, and NC-L; Resources, FW and EM; Supervision, FW and EM; Writing, TG, FW, and EM. All authors read and approved the final manuscript.

*Acknowledgements*

We thank PathGroup Laboratories in Nashville, TN for their generous donation of monoclonal antibodies. We thank Dr. Erin Park (Middle Tennessee State University), Karen Tamul, and David Dickey (Merck Millipore) for flow cytometry training. We also thank both Dr. Erin Park and Jamila Tucker for their phlebotomy expertise. We are appreciative of Dr. Rebecca Seipelt-Thiemann and Dr. David Nelson for their feedback on the manuscript and figures.

**References**

1. Park BJ, Wannemuehler KA, Marston BJ, Govender N, Pappas PG, Chiller TM. Estimation of the current global burden of cryptococcal meningitis among persons living with HIV/AIDS. AIDS. 2009;23(4):525-30.

2. Rajasingham R, Smith RM, Park BJ, Jarvis JN, Govender NP, Chiller TM, et al. Global burden of disease of HIV-associated cryptococcal meningitis: an updated analysis. Lancet Infect Dis. 2017;17(8):873-81.

3. Mora DJ, Fortunato LR, Andrade-Silva LE, Ferreira-Paim K, Rocha IH, Vasconcelos RR, et al. Cytokine profiles at admission can be related to outcome in AIDS patients with cryptococcal meningitis. PLoS One. 2015;10(3):e0120297.

4. Lin YY, Shiau S, Fang CT. Risk factors for invasive *Cryptococcus neoformans* diseases: a case-control study. PLoS One. 2015;10(3):e0119090.

5. Lortholary O, Improvisi L, Fitting C, Cavaillon JM, Dromer F. Influence of gender and age on course of infection and cytokine responses in mice with disseminated *Cryptococcus neoformans* infection. Clin Microbiol Infect. 2002;8(1):31-7.

6. McClelland EE, Hobbs LM, Rivera J, Casadevall A, Potts WK, Smith JM, et al. The role of host gender in the pathogenesis of *Cryptococcus neoformans* infections. PLoS One. 2013;8(5):e63632.

7. Maduro AP, Goncalves L, Inacio J, Faria NC, Martins ML, Teles FR. HIV/AIDS-Associated Cryptococcosis in Portugal Spanning the Pre- to Post-HAART Era: A Retrospective Assessment at the Genotypic Level Based on URA5-RFLP. Curr Microbiol. 2015;71(4):449-57.

8. Richardson ET, Collins SE, Kung T, Jones JH, Hoan Tram K, Boggiano VL, et al. Gender inequality and HIV transmission: a global analysis. J Int AIDS Soc. 2014;17:19035.

9. Shankar J, Wu TD, Clemons KV, Monteiro JP, Mirels LF, Stevens DA. Influence of 17 beta-estradiol on gene expression of Paracoccidioides during mycelia-to-yeast transition. PLoS One. 2011;6(12):e28402.

10. Javed F, Klingspor L, Sundin U, Altamash M, Klinge B, Engstrom PE. Periodontal conditions, oral *Candida albicans* and salivary proteins in type 2 diabetic subjects with emphasis on gender. BMC Oral Health. 2009;9:12.

11. Kali A, Charles MP, Noyal MJ, Sivaraman U, Kumar S, Easow JM. Prevalence of Candida co-infection in patients with pulmonary tuberculosis. Australas Med J. 2013;6(8):387-91.

12. Alves CT, Silva S, Pereira L, Williams DW, Azeredo J, Henriques M. Effect of progesterone on *Candida albicans* vaginal pathogenicity. Int J Med Microbiol. 2014;304(8):1011-7.

13. Tarry W, Fisher M, Shen S, Mawhinney M. *Candida albicans*: the estrogen target for vaginal colonization. J Surg Res. 2005;129(2):278-82.

14. Dromer F, Mathoulin-Pelissier S, Launay O, Lortholary O, French Cryptococcosis Study G. Determinants of disease presentation and outcome during cryptococcosis: the CryptoA/D study. PLoS Med. 2007;4(2):e21.

15. Butts A, Koselny K, Chabrier-Rosello Y, Semighini CP, Brown JC, Wang X, et al. Estrogen receptor antagonists are anti-cryptococcal agents that directly bind EF hand proteins and synergize with fluconazole *in vivo*. MBio. 2014;5(1):e00765-13.

16. Butts A, Martin JA, DiDone L, Bradley EK, Mutz M, Krysan DJ. Structure-activity relationships for the antifungal activity of selective estrogen receptor antagonists related to tamoxifen. PLoS One. 2015;10(5):e0125927.

17. Lizarazo J, Escandon P, Agudelo CI, Castaneda E. Cryptococcosis in Colombian children and literature review. Mem Inst Oswaldo Cruz. 2014;109(6):797-804.

18. Mora DJ, Ferreira-Paim K, Andrade-Silva LE, Bragine T, Rocha IH, Ribeiro BM, et al. Cytokine patterns in a prospective cohort of HIV-infected patients with cryptococcal meningitis following initiation of antifungal and antiretroviral therapy. PLoS One. 2017;12(5):e0176304.

19. Schop J. Protective immunity against *Cryptococcus neoformans* infection. Mcgill J Med. 2007;10(1):35-43.

20. Eschke M, Piehler D, Schulze B, Richter T, Grahnert A, Protschka M, et al. A novel experimental model of *Cryptococcus neoformans*-related immune reconstitution inflammatory syndrome (IRIS) provides insights into pathogenesis. Eur J Immunol. 2015;45(12):3339-50.

21. Li X, Liu G, Ma J, Zhou L, Zhang Q, Gao L. Lack of IL-6 increases blood-brain barrier permeability in fungal meningitis. J Biosci. 2015;40(1):7-12.

22. Rohatgi S, Pirofski LA. Host immunity to *Cryptococcus neoformans*. Future Microbiol. 2015;10(4):565-81.

23. Voelz K, May RC. Cryptococcal interactions with the host immune system. Eukaryot Cell. 2010;9(6):835-46.

24. Aguirre KM, Johnson LL. A role for B cells in resistance to *Cryptococcus neoformans* in mice. Infection and immunity. 1997;65(2):525-30.

25. Rivera J, Zaragoza O, Casadevall A. Antibody-mediated protection against *Cryptococcus neoformans* pulmonary infection is dependent on B cells. Infection and immunity. 2005;73(2):1141-50.

26. Neal LM, Xing E, Xu J, Kolbe JL, Osterholzer JJ, Segal BM, et al. CD4(+) T Cells Orchestrate Lethal Immune Pathology despite Fungal Clearance during *Cryptococcus neoformans* Meningoencephalitis. MBio. 2017;8(6).

27. Lindell DM, Moore TA, McDonald RA, Toews GB, Huffnagle GB. Generation of antifungal effector CD8+ T cells in the absence of CD4+ T cells during *Cryptococcus neoformans* infection. J Immunol. 2005;174(12):7920-8.

28. Vecchiarelli A, Pericolini E, Gabrielli E, Chow SK, Bistoni F, Cenci E, et al. *Cryptococcus neoformans* galactoxylomannan is a potent negative immunomodulator, inspiring new approaches in anti-inflammatory immunotherapy. Immunotherapy. 2011;3(8):997-1005.

29. Nazarpour R, Zabihi E, Alijanpour E, Abedian Z, Mehdizadeh H, Rahimi F. Optimization of Human Peripheral Blood Mononuclear Cells (PBMCs) Cryopreservation. Int J Mol Cell Med. 2012;1(2):88-93.

30. Mody CH, Syme RM. Effect of polysaccharide capsule and methods of preparation on human lymphocyte proliferation in response to *Cryptococcus neoformans*. Infection and immunity. 1993;61(2):464-9.

31. Janbon G, Ormerod KL, Paulet D, Byrnes EJ, 3rd, Yadav V, Chatterjee G, et al. Analysis of the genome and transcriptome of *Cryptococcus neoformans* var. *grubii* reveals complex RNA expression and microevolution leading to virulence attenuation. PLoS Genet. 2014;10(4):e1004261.

32. Stanley J. Essentials of immunology & serology. Albany, NY: Delmar Thomson Learning; 2002. xxi, 538 p. p.

33. Chen LC, Goldman DL, Doering TL, Pirofski L, Casadevall A. Antibody response to *Cryptococcus neoformans* proteins in rodents and humans. Infection and immunity. 1999;67(5):2218-24.

34. Bajnok A, Ivanova M, Rigo J, Jr., Toldi G. The Distribution of Activation Markers and Selectins on Peripheral T Lymphocytes in Preeclampsia. Mediators Inflamm. 2017;2017:8045161.

35. Labatory Reference Ranges: Endocrine Society: Center for Learning; 2015 [cited 2017. Available from: [https://education.endocrine.org/system/files/ESAP 2015 Laboratory Reference Ranges.pdf](https://education.endocrine.org/system/files/ESAP%202015%20Laboratory%20Reference%20Ranges.pdf).

36. Reichert T, DeBruyere M, Deneys V, Totterman T, Lydyard P, Yuksel F, et al. Lymphocyte subset reference ranges in adult Caucasians. Clin Immunol Immunopathol. 1991;60(2):190-208.

37. Valiathan R, Deeb K, Diamante M, Ashman M, Sachdeva N, Asthana D. Reference ranges of lymphocyte subsets in healthy adults and adolescents with special mention of T cell maturation subsets in adults of South Florida. Immunobiology. 2014;219(7):487-96.

38. Edwards VE, Sutherland JM, Tyrer JH. Cryptococcosis of the central nervous system. Epidemiological, clinical, and therapeutic features. J Neurol Neurosurg Psychiatry. 1970;33(4):415-25.

39. Wiesner DL, Moskalenko O, Corcoran JM, McDonald T, Rolfes MA, Meya DB, et al. Cryptococcal genotype influences immunologic response and human clinical outcome after meningitis. MBio. 2012;3(5).

40. Bisson GP, Nthobatsong R, Thakur R, Lesetedi G, Vinekar K, Tebas P, et al. The use of HAART is associated with decreased risk of death during initial treatment of cryptococcal meningitis in adults in Botswana. J Acquir Immune Defic Syndr. 2008;49(2):227-9.

41. Jimenez-Mejias ME, Fernandez A, Alfaro E, Regordan C, Pachon Diaz J. [Cryptococcosis of the central nervous system. Clinical and diagnostic characteristics]. Med Clin (Barc). 1991;97(16):604-8.

42. Manfredi R, Rezza G, Coronado VG, Cozzi Lepri A, Coronado OV, Mastroianni A, et al. Is AIDS-related cryptococcosis more frequent among men? AIDS. 1995;9(4):397-8.

43. Millogo A, Ki-Zerbo GA, Andonaba JB, Lankoande D, Sawadogo A, Yameogo I, et al. [Cryptococcal meningitis in HIV-infected patients at Bobo-Dioulasso hospital (Burkina Faso)]. Bull Soc Pathol Exot. 2004;97(2):119-21.

44. Zheng H, Li M, Wang D, ling Yang J, Chen Q, Zhang X, et al. Gender-specific contributing risk factors and outcome of female cryptococcal meningoencephalitis patients. BMC Infect Dis. 2016;16:22.

45. Vollman RF. The menstrual cycle. Major Probl Obstet Gynecol. 1977;7:1-193.

46. Hajjeh RA, Conn LA, Stephens DS, Baughman W, Hamill R, Graviss E, et al. Cryptococcosis: population-based multistate active surveillance and risk factors in human immunodeficiency virus-infected persons. Cryptococcal Active Surveillance Group. The Journal of Infectious Diseases. 1999;179(2):449-54.

47. Davis J, Zheng WY, Glatman-Freedman A, Ng JA, Pagcatipunan MR, Lessin H, et al. Serologic evidence for regional differences in pediatric cryptococcal infection. Pediatr Infect Dis J. 2007;26(6):549-51.

48. Osazuwa OF, Dirisu O, Okuonghae E. Cryptococcal antigenemia in anti-retroviral naive AIDS patients: prevalence and its association with CD4 cell count. Acta Med Iran. 2012;50(5):344-7.

49. Mohr JA, Muchmore HG, Tacker R. Stimulation of phagocytosis of *Cryptococcus neoformans* in human cryptococcal meningitis. J Reticuloendothel Soc. 1974;15(2):149-54.

50. Roved J, Westerdahl H, Hasselquist D. Sex differences in immune responses: Hormonal effects, antagonistic selection, and evolutionary consequences. Horm Behav. 2017;88:95-105.

51. Colton CA, Brown CM, Vitek MP. Sex steroids, APOE genotype and the innate immune system. Neurobiol Aging. 2005;26(3):363-72.

52. Giefing-Kroll C, Berger P, Lepperdinger G, Grubeck-Loebenstein B. How sex and age affect immune responses, susceptibility to infections, and response to vaccination. Aging Cell. 2015;14(3):309-21.

53. van den Berg MC, Woerlee JZ, Ma H, May RC. Sex-dependent resistance to the pathogenic fungus *Cryptococcus neoformans*. Genetics. 2006;173(2):677-83.

54. Mednick AJ, Nosanchuk JD, Casadevall A. Melanization of *Cryptococcus neoformans* affects lung inflammatory responses during cryptococcal infection. Infection and immunity. 2005;73(4):2012-9.

55. Casadevall A, Pirofski L. Insights into mechanisms of antibody-mediated immunity from studies with *Cryptococcus neoformans*. Curr Mol Med. 2005;5(4):421-33.

56. Ellerbroek PM, Walenkamp AM, Hoepelman AI, Coenjaerts FE. Effects of the capsular polysaccharides of *Cryptococcus neoformans* on phagocyte migration and inflammatory mediators. Curr Med Chem. 2004;11(2):253-66.

57. Szaniawski MA, Spivak AM, Bosque A, Planelles V. Sex Influences SAMHD1 Activity and Susceptibility to Human Immunodeficiency Virus-1 in Primary Human Macrophages. The Journal of Infectious Diseases. 2019;219(5):777-85.

58. Huffnagle GB, Yates JL, Lipscomb MF. Immunity to a pulmonary *Cryptococcus neoformans* infection requires both CD4+ and CD8+ T cells. J Exp Med. 1991;173(4):793-800.

59. Levitz SM, Dupont MP, Smail EH. Direct activity of human T lymphocytes and natural killer cells against *Cryptococcus neoformans*. Infection and Immunity. 1994;62(1):194-202.

60. Panackal AA, Wuest SC, Lin YC, Wu T, Zhang N, Kosa P, et al. Paradoxical Immune Responses in Non-HIV Cryptococcal Meningitis. PLoS Pathog. 2015;11(5):e1004884.

61. Jarvis JN, Casazza JP, Stone HH, Meintjes G, Lawn SD, Levitz SM, et al. The phenotype of the Cryptococcus-specific CD4+ memory T-cell response is associated with disease severity and outcome in HIV-associated cryptococcal meningitis. The Journal of Infectious Diseases. 2013;207(12):1817-28.

62. Rohatgi S, Pirofski LA. Molecular characterization of the early B cell response to pulmonary *Cryptococcus neoformans* infection. J Immunol. 2012;189(12):5820-30.

63. Subramaniam K, Metzger B, Hanau LH, Guh A, Rucker L, Badri S, et al. IgM(+) memory B cell expression predicts HIV-associated cryptococcosis status. The Journal of Infectious Diseases. 2009;200(2):244-51.

64. Garelnabi M, Taylor-Smith LM, Bielska E, Hall RA, Stones D, May RC. Quantifying donor-to-donor variation in macrophage responses to the human fungal pathogen *Cryptococcus neoformans*. PLoS One. 2018;13(3):e0194615.

65. Scriven JE, Graham LM, Schutz C, Scriba TJ, Wilkinson KA, Wilkinson RJ, et al. A Glucuronoxylomannan-Associated Immune Signature, Characterized by Monocyte Deactivation and an Increased Interleukin 10 Level, Is a Predictor of Death in Cryptococcal Meningitis. The Journal of Infectious Diseases. 2016;213(11):1725-34.

66. Van Dyke MCC, Chaturvedi AK, Hardison SE, Leopold Wager CM, Castro-Lopez N, Hole CR, et al. Induction of Broad-Spectrum Protective Immunity against Disparate Cryptococcus Serotypes. Front Immunol. 2017;8:1359.

67. Zhang X, Lv X, Song Y. Short-term culture with IL-2 is beneficial for potent memory chimeric antigen receptor T cell production. Biochem Biophys Res Commun. 2018;495(2):1833-8.

68. Lehmann C, Zeis M, Uharek L. Activation of natural killer cells with interleukin 2 (IL-2) and IL-12 increases perforin binding and subsequent lysis of tumour cells. Br J Haematol. 2001;114(3):660-5.

69. Mohr JA, Long H, McKown BA, Muchmore HG. In vitro susceptibility of *Cryptococcus neoformans* to steroids. Sabouraudia. 1972;10(2):171-2.

70. Abadi J, Pirofski L. Antibodies reactive with the cryptococcal capsular polysaccharide glucuronoxylomannan are present in sera from children with and without human immunodeficiency virus infection. The Journal of Infectious Diseases. 1999;180(3):915-9.

71. Census US. Murfreesboro, TN. V2016 ed. Retrieved from: <https://www.census.gov/quickfacts/fact/table/murfreesborocitytennessee/PST0452162016>.

72. Angele MK, Pratschke S, Hubbard WJ, Chaudry IH. Gender differences in sepsis: cardiovascular and immunological aspects. Virulence. 2014;5(1):12-9.

73. Imahara SD, Jelacic S, Junker CE, O'Keefe GE. The influence of gender on human innate immunity. Surgery. 2005;138(2):275-82.

74. Candore G, Balistreri CR, Listi F, Grimaldi MP, Vasto S, Colonna-Romano G, et al. Immunogenetics, gender, and longevity. Ann N Y Acad Sci. 2006;1089:516-37.

75. Kim-Fine S, Regnault TR, Lee JS, Gimbel SA, Greenspoon JA, Fairbairn J, et al. Male gender promotes an increased inflammatory response to lipopolysaccharide in umbilical vein blood. J Matern Fetal Neonatal Med. 2012;25(11):2470-4.

76. Guess T, McClelland E. An Updated Overview of the Gender-Specific Response to Infection. Principles of Gender-Specific Medicine 3rd ed. Cambridge: Elsevier, Inc.; 2017. p. 417-28.

77. Sofronescu A. Estradiol Reference Range: MedScape; 2015 [Available from: <https://emedicine.medscape.com/article/2089003-overview?pa=L4udVARM1XDHbdrQzqXYLMdFqfh6oL2AJ3pzG%2FHCwYXR1ciqbewlOcfnwAsAfTYsgqoa090UYJ8Fd8FkU1fbR%2FEiL5fM42L%2B9xlMlua7G1g%3D>.

78. Berthois Y, Katzenellenbogen JA, Katzenellenbogen BS. Phenol red in tissue culture media is a weak estrogen: implications concerning the study of estrogen-responsive cells in culture. Proc Natl Acad Sci U S A. 1986;83(8):2496-500.

79. Milo GE, Malarkey WB, Powell JE, Blakeslee JR, Yohn DS. Effects of steroid hormones in fetal bovine serum on plating ang cloning of human cells in vitro. In Vitro. 1976;12(1):23-30.

**Figure Legends**

**Figure 1. Experiment and workflow schematic.** Whole blood was extracted by venipuncture and separated using a density gradient. Isolated PBMCs were incubated for 7 days either alone or in the presence of *C. neoformans*. Subsets of both uninfected and infected groups were incubated with testosterone or 17 β-estradiol. All samples were setup in triplicate and the B and T cell response was determined via flow cytometry in the first dataset. The T cell and natural killer cell response was determined in the second dataset. Dead cells were identified in both datasets using dead cell stain. Because no differences were observed in cells incubated with sex hormones, only cell supernatants of uninfected and infected PBMCs (without hormones), from the first dataset, were used for cytokine analysis via Luminex assay.

**Figure 2. Male T Cell Dot Plots.** Dot plots from a representative male donor (2017-45), in the first dataset, of CD3^+^/CD4^+^ and CD3^+^/CD8^+^ T cells in both uninfected and infected treatment groups.

**Figure 3. Female T Cell Dot Plots.** Dot plots from a representative female donor (2017-14), in the first dataset, of CD3^+^/CD4^+^ and CD3^+^/CD8^+^ T cells in both uninfected and infected treatment groups.

**Figure 4. *In situ* cytokine analysis and CD25 levels**. (A) Cytokine levels from infected male PBMCs: IL-2, IL-4, IL-6, IFN-γ, and TNF-α compared to uninfected PBMCs. N = 40 (21 males & 19 females). Cytokine analysis was done on the first data set using one well of the triplicate samples set up (no additional hormones) and measured using the Bio-Plex protein array or ELISA, and the data was analyzed using a two-way ANOVA and Bonferroni Correction. Error bars are SE. Statistical significance is indicated as follows: **p* < 0.05. (B) Levels of CD25^+^ cells between male and female uninfected and infected PBMCS after 7 days of infection as measured by cytometric analysis. The total percentage given represents the total percentage of cells expressing CD25^+.^ N = 5 (3 males & 2 females). This experiment was done in triplicate and the results averaged to create each data point. Data was analyzed using MANOVA with simple contrasts. Error bars are SE; n.s. = not significant.

**Figure 5. Differences in immune response between sexes in uninfected PBMCs (first dataset).** Mean cell percentages for the T cell markers, **(A)** CD3^+^, **(B)** CD4^+^, **(C)** CD8^+^, and **(D)** the B cell marker, CD19^+^, in PBMCs from healthy men and women after a 7 day incubation as measured by cytometric analysis. The total percentage given for each marker represents the total percentage of cells expressing the antigen indicated. N = 40 (21 males & 19 females). This experiment was done in triplicate and the results averaged to create each data point. Data was analyzed using MANOVA with simple contrasts. Error bars are SE; n.s. = not significant.

**Figure 6. Differences in immune response between sexes in uninfected PBMCs (second dataset).** Mean cell percentages for the T cell marker, **(A)** CD3^+^ and the natural killer cell marker **(B)** CD56^+^, in PBMCs from healthy men and women after a 7 day *C. neoformans* incubation as measured by flow cytometric analysis. The total percentage given for each marker represents the total percentage of cells expressing the antigen indicated. N = 28 (14 males & 14 females). This experiment was done in triplicate and the results averaged to create each data point. Data was analyzed using MANOVA with simple contrasts. Error bars are SE; n.s. = not significant.

**Figure 7*. C. neoformans* proliferation at 7 days post-infection.** Mean cell counts of *C. neoformans* as measured by flow cytometry after 7 days incubation with peripheral blood mononuclear cells isolated from healthy men or women in the presence of **(A)** no additional hormones, **(B)** physiological levels of testosterone, or **(C)** physiological levels of estrogen. N = 28 (14 males & 14 females). Error bars are standard error; n.s. = not significant.

**Figure 8. Differences in immune response between sexes in infected PBMCs (first dataset).** Mean cell percentages for the T cell markers, **(A)** CD3^+^, **(B)** CD4^+^, **(C)** CD8^+^, and **(D)** the B cell marker, CD19^+^, in PBMCs from healthy men and women after a 7 day *C. neoformans* infection as measured by cytometric analysis. The total percentage given for each marker represents the total percentage of cells expressing the antigen indicated. N = 40 (21 males & 19 females). This experiment was done in triplicate and the results averaged to create each data point. Data was analyzed using MANOVA with simple contrasts. Error bars are SE; n.s. = non-significant.

**Figure 9. Differences in immune response between sexes in infected PBMCs (second dataset).** Mean cell percentages for the T cell marker, **(A)** CD3^+^ and the natural killer cell marker **(B)** CD56^+^, in PBMCs from healthy men and women after a 7 day *C. neoformans* infection as measured by flow cytometric analysis. The total percentage given for each marker represents the total percentage of cells expressing the antigen indicated. N = 28 (14 males & 14 females). This experiment was done in triplicate and the results averaged to create each data point. Data was analyzed using MANOVA with simple contrasts. Error bars are SE; n.s. = non-significant.

**Figure 10. Cytokine analysis of male and female PBMCs supernatants (pg/mL) at 7 days post-incubation**. **(A)** Th1 cytokine expression and **(B)** Th2 cytokine expression N = 40 (21 males & 19 females). Cytokine analysis was done on the first data set using one well of the triplicate samples set up (no additional hormones) and measured using the Bio-Plex protein array or ELISA, and the data was analyzed using a two-way ANOVA and Bonferroni Correction. Error bars are SE. Statistical significance is indicated as follows: *, *p* < 0.05; **, *p* < 0.01; and ***, *p* < 0.001.

**Figure 11. Cytokine analysis of uninfected and infected cell supernatants (pg/mL) at 7 days post-incubation. A.** Th1 cytokines **B.** Th2 Cytokines. N = 40 (21 males & 19 females). Cytokine analysis was done on the first data set using one well of the triplicate samples set up (no additional hormones) and measured using the Bio-Plex protein array or ELISA, and the data was analyzed using a two-way ANOVA and Bonferroni Correction. Error bars are SE. Statistical significance is indicated as follows: *, *p* < 0.05; **, *p* < 0.01; and ***, *p* < 0.001.

Figure 1


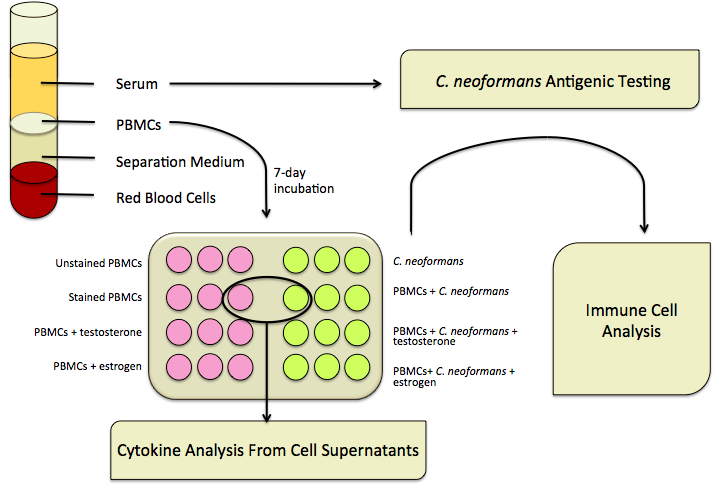


Figure 2


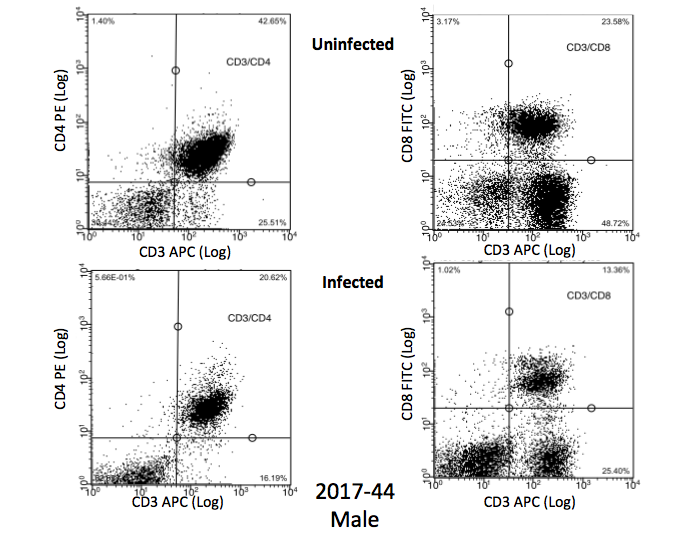


Figure 3


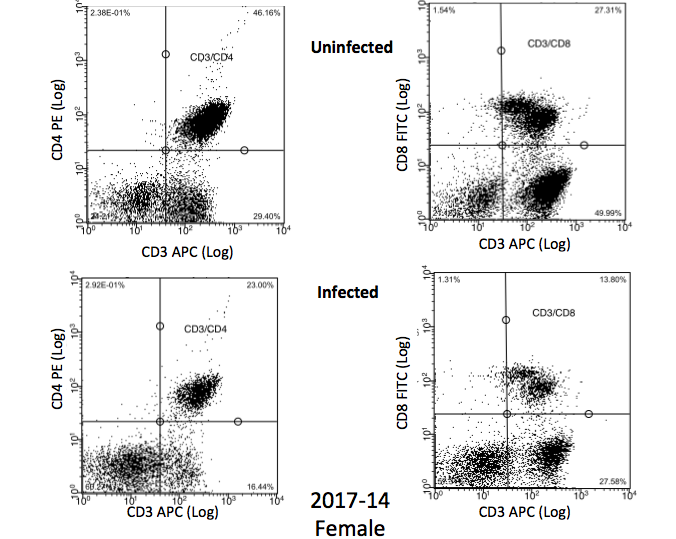


Figure 4.


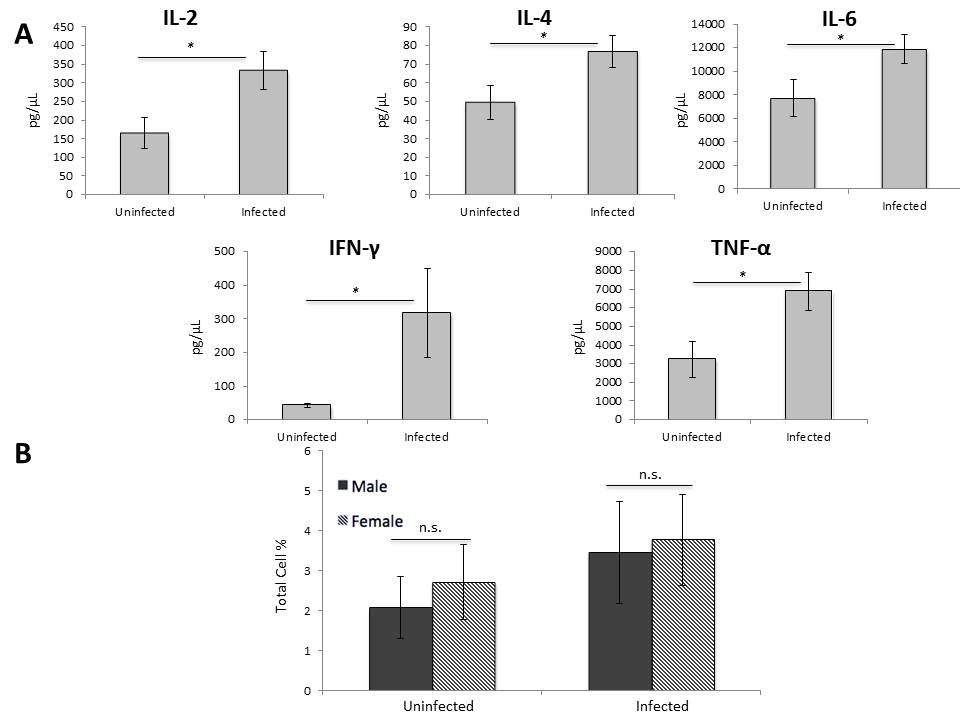


Figure 5


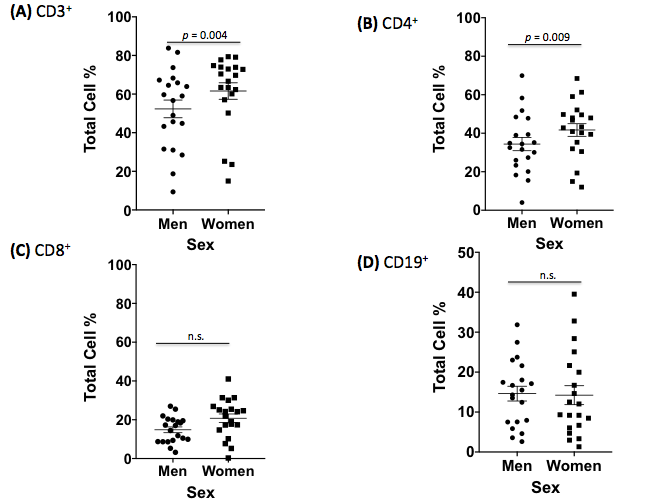


Figure 6


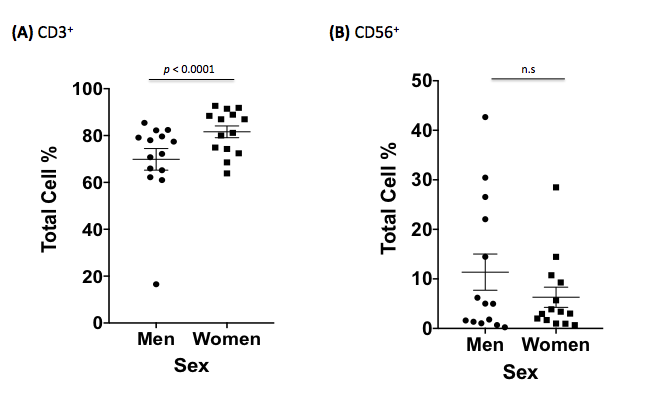


Figure 7


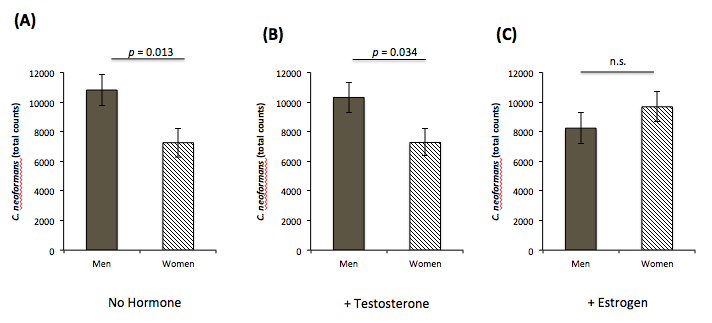


Figure 8


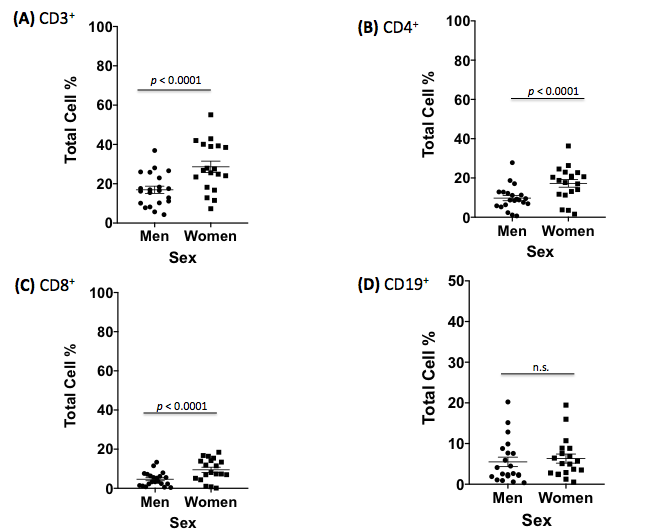


Figure 9


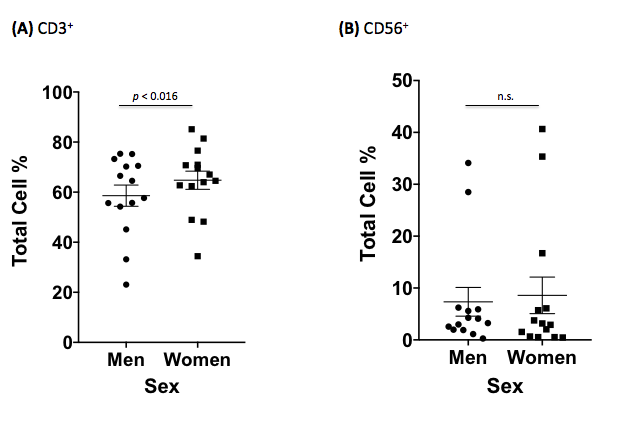


Figure 10


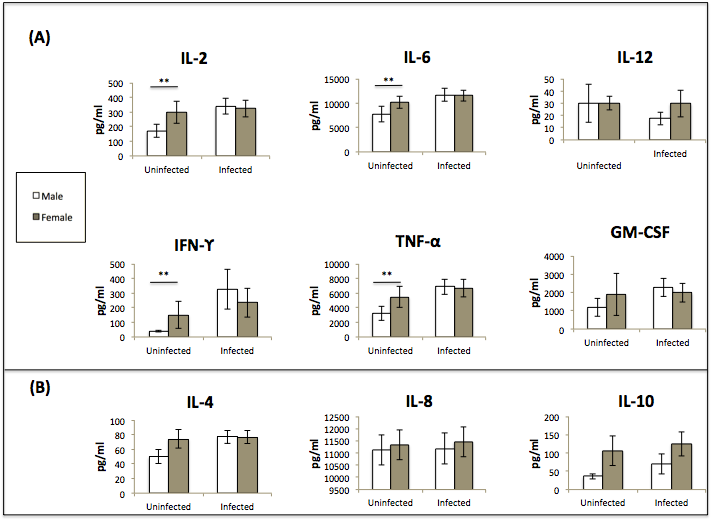


Figure 11


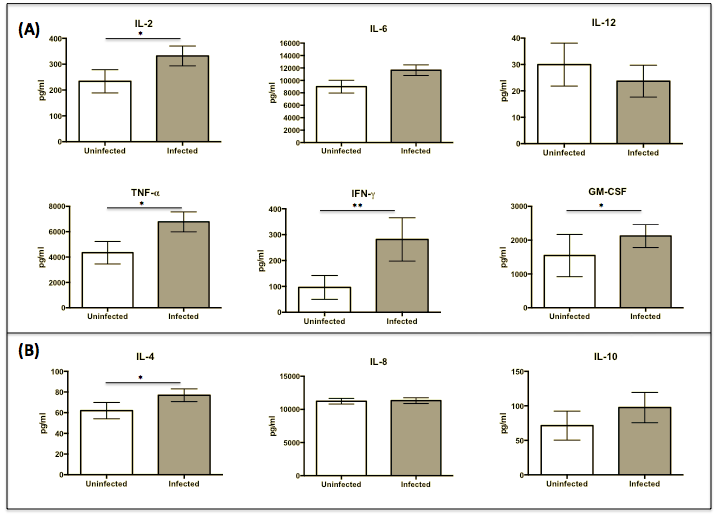

Supplement: Supplementary file 2 — Flow cytometry cell counts for second dataset (DOCX 1010 kb) [file 13293_2019_258_MOESM2_ESM.docx]
